# Recommendations for the Inclusion of Autistic Children in Community-Based Physical Activity Programmes: A Delphi Study

**DOI:** 10.1177/13623613261448516

**Published:** 2026-05-31

**Authors:** Edel Ryan, Dean McDonnell, Sean Healy, Rhodri S. Lloyd, Sharon Kinsella

**Affiliations:** 1 South East Technological University, Ireland; 2 University of Limerick, Ireland; 3 Cardiff Metropolitan University, Cardiff, UK; 4 Auckland University of Technology, New Zealand; 5 Waikato Institute of Technology, New Zealand

**Keywords:** adaptive physical activity, autism, bridging sessions, coach education, exercise, STARTS recommendations

## Abstract

A range of barriers to Physical Activity (PA) participation contribute to autistic children being less physically active than their non-autistic peers. While these barriers have been well documented, further research is needed into methods of overcoming these barriers. This study aimed to create a series of best practice recommendations for the inclusion of autistic children in community-based PA programmes. This was achieved using the Delphi Method involving the opinions of international experts in the area of inclusion of autistic children in PA. Three rounds of questionnaires were distributed to the panel of 22 experts, aiming to reach consensus on a series of topics related to the inclusion of autistic children in community-based PA programmes (consensus ⩾67% agreement). Topics included coaches and volunteers, programme characteristics, and bridging sessions. Sixteen consensus statements were established which were then used to create the ‘STARTS’ recommendations, which centre around: (1) Skill development, (2) Training for coaches/volunteers, (3) Aims for sessions, (4) Resources to improve children’s experiences, (5) Transitioning from bridging programmes to mainstream programmes, and (6) Supports to improve inclusion. The STARTS recommendations will assist clubs/organisations when setting up or modifying community-based PA programmes which include autistic children.

**Lay abstract:**

Autistic children can often find participation in physical activity programmes difficult and as a result are often less physical active than to non-autistic children. Many studies have explained why these difficulties exist, such as lack of suitable programmes, uneducated coaches, lack of coach, or physicality difficulties, but there is less information available about practical ways to make programmes more inclusive. This study aimed to create clear, practical recommendations to help community sports clubs and physical activity programmes to better include autistic children. To do this, the Delphi Method was used. This involved a group of ‘experts’ with experience in autism and physical activity to share their views over several rounds of online questionnaires. Twenty-two international experts took part in this study, and over three rounds, they were asked to share suggestions and opinions, answer multiple-choice questions, and rate different ideas. By the end of the process, the experts agreed on 16 key recommendations, which were then used to create the ‘STARTS’ recommendations. This splits the recommendations into six topics: (1) Skill development, (2) Training for coaches/volunteers, (3) Aims for sessions, (4) Resources to improve children’s experiences, (5) Transitioning from autism-specific programmes to mainstream programmes, and (6) Supports to improve inclusion. These recommendations offer practical guidance for clubs and organisations that want to create or adapt community-based physical activity programmes to promote inclusion and help autistic children feel welcomed and supported.

## Introduction

The global prevalence of autism is reported to be approximately 1 in 100 children (Zeidan et al., 2022); however, figures vary significantly across countries. For example, in the United States, the prevalence of autism among 8-year-old children is estimated to be around 1 in 31 (Shaw, 2025), while in Northern Ireland, recent reports estimate figures to be 1 in 17 children (Kinghan & Pham, 2025). Autistic children generally partake in less Physical Activity (PA) in comparison to their non-autistic peers (Case et al., 2020; Healy et al., 2017; Rech et al., 2022; Stanish et al., 2017) and are less physically fit in comparison to age-matched, non-autistic peers (Coffey et al., 2021). Stanish et al. (2017) reported that up to 86% of autistic adolescents did not meet the recommended American PA guidelines, of 60 minutes of moderate-to-vigorous intensity PA (USDHHS, 2008), which is consistent with more recent research using accelerometer-measured data (Liang et al., 2020). While poor motor skills are not considered a key indicator of autism, there is a growing body of evidence suggesting that autistic children commonly present with motor skill impairments (Licari et al., 2022; Whyatt & Craig, 2012) and do not develop their fundamental movement skills (FMSs) at the same rate as their non-autistic peers (Gandotra et al., 2020; Liu & Breslin, 2013).

Research has shown that PA elicits positive effects on autism characteristics, such as improvements in communication and social functioning (Chan et al., 2021; Healy et al., 2018; Howells et al., 2019; Hynes & Block, 2023) and decreasing stereotypical or self-harming behaviours (Bahrami et al., 2012; Movahedi et al., 2013). In addition, PA has shown improvements in autistic children’s fine and gross motor skills (Busti Ceccarelli et al., 2020; Case & Yun, 2019; Colombo-Dougovito & Block, 2019; Pan, 2011), skill-related fitness, muscular strength and endurance (Healy et al., 2018; Pan, 2011; Todd et al., 2010), and increased focus and cognitive functioning (Anderson-Hanley et al., 2011; Hynes & Block, 2023). Moreover, participation in group-based PA programmes can provide further opportunities for social and communication skill development for autistic children (Howells et al., 2019).

While the benefits of PA for the autistic population have been widely reported within the literature, autistic children still face an extensive range of barriers to PA participation (Okkenhaug et al., 2024). Parent-reported research concluded that autistic children face significantly more barriers to PA in comparison to non-autistic peers, with around 51% of parents of autistic children reporting ⩾6 barriers (Must et al., 2015). Common barriers included poor motor skills, behavioural and learning problems, and community-related barriers pertaining to cost, unavailability of suitable programmes and transportation. In addition, barriers relating to communication and socialisation are reported to make participation in structured PA programmes with non-autistic peers challenging (Must et al., 2015; Obrusnikova & Miccinello, 2012). Families with autistic children commonly only have access to mainstream community-based programmes that often lack the necessary support, training, and resources to support autistic children (Obrusnikova & Cavalier, 2011). The perspectives of autistic children have also been explored, with child-reported barriers including limited availability of suitable community programmes, experiences of exclusion and bullying from peers and a tendency for parents to prioritise therapeutic interventions (Jachyra et al., 2021). Similarly, research exploring parents’ perspectives of PA participation for autistic children also reported prioritisation of behavioural and communication interventions over PA (Gregor et al., 2018).

A recent scoping review by Okkenhaug et al. (2024) identified several key facilitators to autistic children’s PA participation. Family support was reported to be an important factor, with prioritisation of PA and financial support being key for participation. Similarly, competent coaches with qualities such as understanding, humour, and effective communication skills were cited as important factors for the facilitation of PA. Additional facilitators reported related to the availability of inclusive programmes, personal motivation to participate, predictability and structure of programmes and social competence. Although facilitators to PA have been identified within the literature, there remains limited research examining the practical applications which can be made in community-based settings.

Research regarding PA interventions for autistic children is often conducted through universities in controlled settings, which usually have access to research funding, educated researchers, and research assistants (Pan et al., 2017). Due to the typically controlled procedures used within these studies, generalising the findings to a community-based context is not always possible (Leichsenring, 2004) because of barriers relating to costs, facilities, staff, and resources (Miller et al., 2012). Community-based programmes, which are generally a more natural environment for autistic children, have the potential for increased socialisation opportunities (Howells et al., 2019) and, therefore, may improve social functioning. However, to the best of the authors’ knowledge, there are currently no inclusion recommendations for community-based PA programmes for autistic children, which have been informed through expert opinion.

Considering the current limitations within the literature, this Delphi study aimed to utilise the knowledge and opinions of ‘experts’ to develop a series of autism inclusion recommendations for children’s community-based PA programmes. These proposed recommendations could subsequently be used by coaches, organisations, or practitioners when setting up or modifying community-based PA programmes that would successfully include autistic children. Despite the growing research on autism inclusion in PA, a large amount of the research focuses on highlighting the barriers and facilitators to PA participation. This study aims to add to this area of research by creating feasible and practical recommendations to overcome the previously established barriers in a community-based context. The outcome of this study has the potential to improve the experiences of autistic children and their families in community-based PA programmes if recommendations are adopted by clubs and organisations.

## Method

The Delphi Method (Linstone & Turoff, 1975) is a structured process which aims to collate the ideas and opinions of an international panel of ‘experts’ in a specific area to come to a general consensus on a given issue. The Delphi Method was chosen as the approach for the current study because it allowed for the combination of the knowledge of an international panel of experts on the inclusion of autistic individuals within sports and autistic individuals who have experience of participating in sports. In addition, this method was chosen because its anonymous nature allowed participants to give honest opinions freely without fear of judgement from others, while also allowing participants opportunities to think and reflect on other participants’ opinions. The panel of experts was required to complete a series of questionnaires, with the results of each round collated and used to formulate the subsequent round. This method was followed until a consensus was reached for each topic. Ethical approval for this research was granted by the South East Technological University Research Ethics Committee (SETU/REC/22/23/373).

### Recruitment and Participants

The sample for this study included (a) academics who had published on the topic of community-based or group PA programmes for autistic children, (b) professionals working/coaching in the area of the inclusion of autistic children in community-based or group PA programmes, and (c) autistic individuals who had experience either coaching autistic children in PA programmes or had experience being an athlete themselves. Potential participants were identified via: (a) searches of electronic databases to find suitable academics with relevant peer-reviewed journal publications, (b) internet searches of various sporting bodies/organisations who could be contacted to identify suitable inclusion officers or coaches, (c) autism advocacy groups who were contacted and asked to identify suitable working professionals and autistic individuals, (d) the associated university’s Autism Research Group network, and (e) recommendations of suitable personnel from other experts.

**Figure 1.**
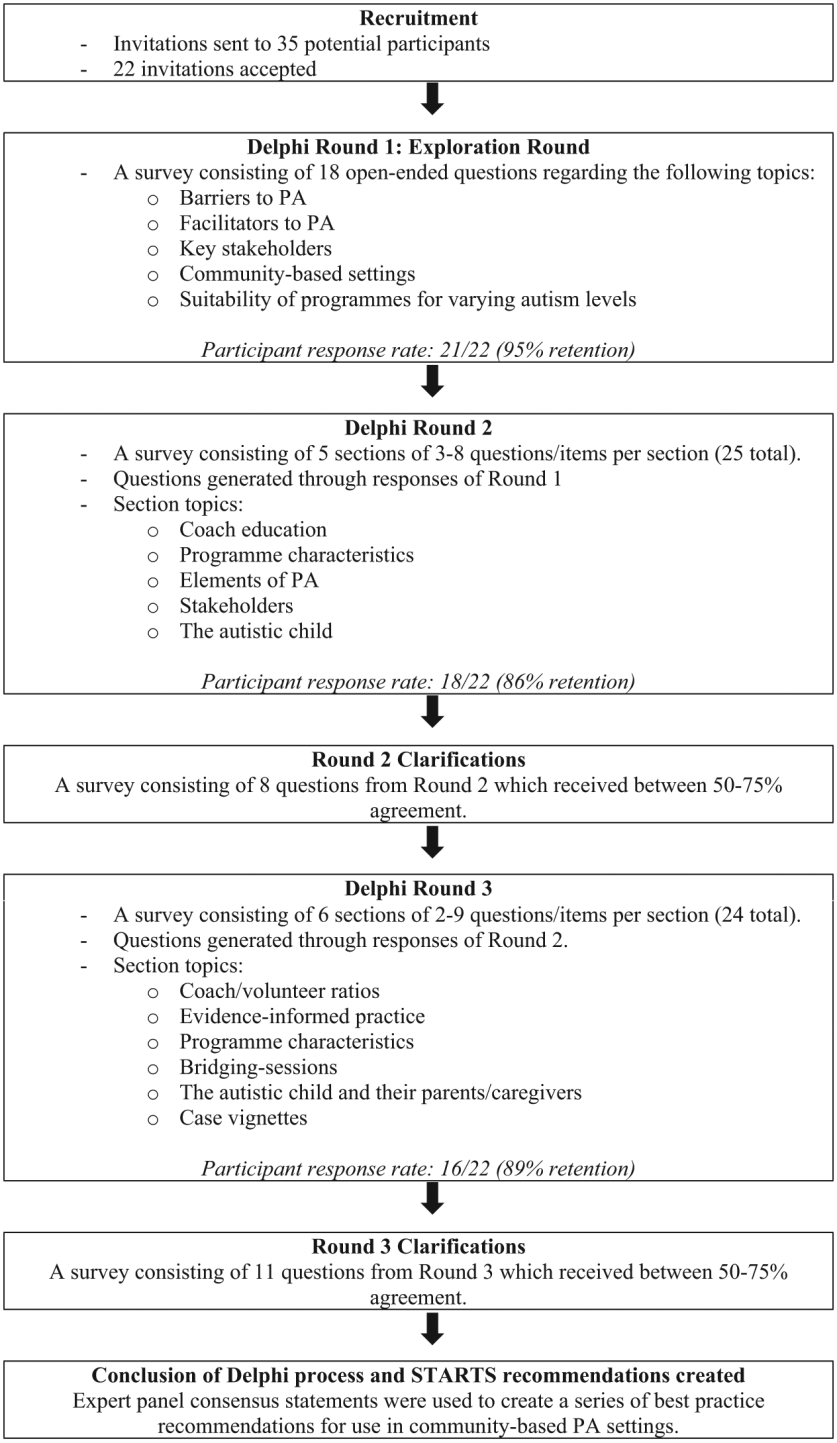
Summary of the Delphi process. PA: physical activity.

Invitations, including a description of the study and the expected time requirements, were sent via email to a total of 35 individuals, of which 22 agreed to participate (12 females and 10 males). Within the final sample, 13 participants had an academic background, seven were working professionals, and two were autistic individuals. The sample included 16 Europeans, four Americans, and two Australians. Participant retention rates for each round are displayed in Figure 1.

### Delphi Process

The Delphi study was conducted via the online survey platform, Qualtrics (Qualtrics, Provo, Utah, USA). The study spanned a 3-month period, and the results of each round were used to guide and structure the subsequent rounds (Figure 1). Round 1 began with 18 open-ended questions which were deemed relevant to the research question by the primary author and were screened by all co-authors. Questions for the subsequent round were based on participant responses and created by the primary author and screened by all co-authors. Notably, two of the co-authors would have met the inclusion criteria for the expert panel. From the release day, participants had 1 week to complete each questionnaire. This timeframe was chosen to allow adequate time for completion while maximising retention and engagement between rounds (Keeney et al., 2010). Participants received two reminders to complete the questionnaire during this week. Within the following 2 weeks, the responses were analysed and used to create the subsequent round. Rounds consisted of open- and closed-ended questions/items, and each round was estimated to take approximately 30 minutes to complete. Following the completion of each round, participants received a ‘summary of results’ report. The reports outlined the frequency of themes generated through open-ended questions and frequencies or percentage agreements for close-ended questions/items. All results were reported anonymously, allowing panellists to reflect on their responses in comparison to fellow experts without fear of their own opinions being viewed negatively. Before the distribution of each round, questions were screened by the primary author and all co-authors.

### Consensus Criteria and Clarifications

As the likelihood of gaining complete agreement on topics across an expert panel is very low, a ‘consensus’ was defined as a topic reaching ⩾67% agreement (Von Der Gracht, 2012). Consensus could be achieved by ⩾67% of the panellists selecting a specific item on a multiple-choice or rating scale, or by selecting ‘strongly agree’ or ‘agree’ on Likert-type scale items (Devereux et al., 2024; Gavigan et al., 2022; McCall et al., 2020). To ensure the stability of responses from experts, clarification rounds were used in the case where a topic received between 50% and 75% agreement (Devereux et al., 2024). In cases where questions required further clarification, they were sent back to experts, with the existing agreement levels (e.g., ‘61.6% of experts said . . .’) presented. Clarification rounds were sent out 1 week after the response deadline, enabling experts to reflect on the topic and ensure the accuracy of the responses was captured within the process. Once clarification rounds were completed, a final list of topics where consensus was found was established.

### Round 1: Exploration Round

An initial exploration round was conducted to gather a wide range of themes and ideas, which were used to generate topics for the subsequent rounds. This round consisted of six sections, with a total of 18 open-ended questions which aimed to explore the panel of experts’ opinions on the: (a) barriers to PA among autistic children, (b) factors which support the participation of autistic children in PA, (c) key stakeholders who are necessary to facilitate this participation, (d) characteristics of successful/unsuccessful autism inclusive community-based programmes, and (e) suitability of a community-based programme for autistic children with varying symptom levels. This round did not require a clarification round because the questions were open-ended.

### Rounds 2 and 3

Responses from the exploration round were coded and thematically analysed by the primary author and reviewed by one of the co-authors. Commonly cited themes/topics were established and used to create five sections in which to explore in Round 2. Each section consisted of questions/items generated by the primary author within the themes generated from the previous round. All questions/items were screened by co-authors before rounds went live. Questions/items were mainly asked in the form of five-point Likert-type Scales (e.g., ‘very important’ to ‘not important at all’), multiple choice, or rankings (e.g., rank in order of importance). Eight open-ended questions were also included in this round where relevant, four of which asked participants if they had anything else relevant, they would like to add in relation to a certain topic. In the case of conflicting recommendations from Round 1, both suggestions were presented to the panellists in the form of Likert-type scales. When the analysis of Round 2 was completed, consensus was found in several topics, while others required further exploration. The development of Round 3 followed the same methods. Similarly, this round consisted mainly of Likert-type scales, multiple choice or rankings, with three additional open-ended questions.

### Data Analysis

The responses from each round were retrieved from the Qualtrics software. The software generated descriptive statistics, including means, frequencies, and percentage agreement data in cases of close-ended questions/items (e.g., Likert-type scales, rankings, or multiple choice). In the case of open-ended questions, responses were extracted verbatim for qualitative analysis. NVivo v.12 software (QRS International, Doncaster, Australia) was used to thematically analyse the data using the six-step approach outlined by Braun and Clarke (2006); (1) data familiarisation, (2) generating primary codes, (3) examining for themes, (4) reviewing generated themes, (5) defining themes, and (6) final analysis report. An inductive approach was used during the analysis of Round 1 data to generate themes and sub-themes, which were then used to create content for the subsequent rounds. This approach allowed for themes and ideas to emerge naturally from the data and ensured that the content for subsequent rounds was grounded in the participants’ personal experiences and expertise (Fereday & Muir-Cochrane, 2006; Naeem et al., 2023). Open-ended questions in Rounds 2 and 3 were optional opportunities for experts to further comment or provide clarification on closed-ended questions from a particular section of the survey. There were typically very few responses for these, and responses were generally short. Therefore, open-ended questions in Rounds 2 and 3 were analysed by hand and any new themes or relevant ideas, as deemed by the research team, were included in the following round. No new themes or relevant ideas were identified in the open-ended questions of Round 3; therefore, a subsequent round was deemed unnecessary.

## Results

The Delphi process generated a series of consensus statements that provide insight into practical recommendations for PA programmes to include autistic children. These recommendations centre around three main themes: (1) coaches and volunteers, (2) programme characteristics, and (3) bridging sessions. These results were then used to create the ‘STARTS’ recommendations, which break the themes down into (1) Skills, (2) Training, (3) Aims, (4) Resources, (5) Transition, and (6) Support (see Supplementary Material for full list of recommendations).

### Coaches and Volunteers

#### Round 1

Coaching-related factors were the most cited barriers which hinder the inclusion/participation of autistic children in community-based PA programmes [mentions (*n*) = 28]. These factors were related to issues with coaching methods, coach education, and lack of coaching staff, with one expert stating that a *‘lack of knowledge of community staff on how to work with children with autism’* was a primary barrier.

Participants outlined how barriers could be directly addressed, and the need for high quality, autism-specific coach education was again the most cited answer (*n* = 19). Specifically, methods of inclusion, behavioural strategies, the Picture Exchange Communication System or ‘PECS’, sensory integration, and Fundamental Movements Skills (FMS), were the areas primarily identified as a means of addressing barriers. Similarly, when asked about the factors or considerations which are essential for supporting autistic children in community-based PA programmes, answers relating to coaches (i.e., coach education, number of coaches, attitudes of coaches) were the second most cited topic by experts (*n* = 17). When asked about the factors that make a community-based setting suitable for an autistic child, factors relating to coaches or volunteers were cited most frequently (*n* = 12), followed by factors relating specifically to coach-to-child ratios (*n* = 8).

#### Round 2

Round 2 aimed to explore the topic of coach education further, with consensus being reached on the necessity of a coach working with autistic children having education in the areas of: (1) methods of inclusion (100% agreement), (2) behavioural strategies (94.4% agreement), (3) knowledge and delivery of FMS (94.4% agreement), (4) sensory integration (88.9% agreement), and (5) use of the Picture Exchange Communication System (75% agreement). In relation to the topic of coach education, one expert highlighted the inadequacies of current training offerings, noting: *‘. . . current education is more of an awareness rather than education’*, which suggests a possible need for a review of current autism in sport coach education.

The expert panel was asked about the facilitation of training. Consensus was reached on the following statements: ‘It is the role of the management of a sports club to ensure their coaching staff are educated in autism-specific training’ (88.9% agreement) and ‘it is the role of governing sporting bodies to ensure that autism in sport training is rolled out to individual clubs’ (94.4% agreement). An agreement was not reached concerning whether the individual coach should be responsible for seeking additional autism-specific training themselves. Experts also agreed that coaches should complete refresher courses to stay up-to-date on current research recommendations (77.8% agreement) and that training workshops should be offered to community members (100% agreement).

#### Round 3

Although 83.4% of experts agreed with the statement that ‘programmes should be modelled around evidence-based research’ in Round 2, there was only 62.25% agreement (non-consensus) with the statement ‘coaches should have access to evidence-based research and should be implementing the research into their programmes’. These differing responses may be due to an issue with knowledge transfer, with one expert stating: *‘there is a wide variety of educational levels within coaching communities so having direct access to the research may not work for all coaches’*. Consensus was reached for several statements regarding the importance of evidence-informed practice and knowledge transfer (Table 1).

**Table 1.** Evidence-Informed Practice and Knowledge Transfer-Related Consensus Statements (Consensus ⩾67% Agreement).

Statement	Agreement
‘Coaches should seek out research to better inform their programmes, which include autistic children’	91.67%
‘Autism inclusion coaching courses should include the most up-to-date research to allow coaches to design evidence-informed programmes’	88.2%
‘Autism inclusion coaching courses/training should provide newsletters/online resources to previous clients, which outline the most up-to-date research, so coaches can continue to create evidence-informed programmes’	82.4%
‘Governing sporting bodies should provide newsletters/online resources which outline the most up-to-date research, so coaches can continue to create evidence-informed programmes’	82.3%
‘National authorities which oversee the development of sport should provide newsletters/online resources which outline the most up-to-date research, so coaches can continue to create evidence-informed programmes’	88.2%

The need for volunteers was commonly cited throughout the first two rounds. Round 3 sought to establish the requirements for volunteers and the ideal ratios of volunteers to autistic children. The panel reached consensus on several statements (Table 2). It should be noted that 50% of experts said that volunteers should not be expected to complete autism-specific training. Experts were asked about volunteer-to-child ratios within the context of one educated coach being present. The panel did not reach consensus for this question, and mean scores for each volunteer-to-child ratio ranged considerably; as a result, averages are given. For four autistic children – 1.8 volunteers; six autistic children – 3.1 volunteers; eight autistic children – 4.1 volunteers and ten autistic children – 5.2 volunteers. Average responses suggest that volunteer-to-child ratios should be approximately 1:2.

**Table 2.** Volunteer Involvement Consensus Statements (Consensus ⩾67% Agreement).

Statement	Agreement (%)
*Volunteers must . . .*	
. . . be educated in methods of inclusion of autistic children	82.3
. . . be educated in behavioural strategies	82.3
. . . be educated in sensory education	75
. . . understand FMS and how to develop them	75
. . . complete refresher courses to stay up-to-date on current best practice guidelines for the inclusion of autistic children in PA programmes	75

FMS: fundamental movement skills; PA: physical activity.

### Programme Characteristics

#### Round 1

Factors relating to programme characteristics were the second most cited barrier to participation by experts (*n* = 21). Programme characteristics mentioned included resources required for sessions, the length/frequency of sessions, the organisational aspect of a programme, the costs of these programmes, and coach/volunteer availability. Child-centred factors pertaining to physical literacy, communication deficits, and lack of enjoyment/interest in PA were also cited by several experts (*n* = 8). Participants were asked how these barriers could be addressed, and amendments to current programmes were frequently cited (*n* = 18). When asked about the factors/considerations that are important for supporting autistic children in community-based PA programmes, answers relating to programme characteristics were the most frequently cited by experts (*n* = 25). Similarly, when asked about the factors which make a community-based setting suitable for an autistic child, programme characteristics were commonly cited (*n* = 19), with the majority pertaining to the physical venue in which a programme should take place (*n* = 16). For example, in relation to the suitability of a setting, one expert noted that: *‘There are no unsuitable settings if preparation is carried out in advance . . . a venue that is large and echoey with bright lights can be adapted . . . reduce scale to smaller sub-zones . . .’.*

#### Round 2

For a successful community-based PA programme, a number of factors were considered necessary, including a specific area for children when they need a break, a visual explanation of how each session will run, visual aids for each game/exercise in the form of pictures, a variety of sports equipment for adapting games (all 100% agreement), iPad/tablets with demonstration videos as visual aids for each game/exercise (80% agreement), and a timer for children (80% agreement).

The ideal individual session length was deemed to be 30–45 minutes, with 86.67% of experts selecting this to be optimal, while twice weekly was considered the best session frequency (68.75% agreement). Consensus was also found for the following: ‘if an autistic child does not want to participate in a PA programme, they should be allowed to sit out immediately’ (75% agreement); however, in contrast, 77.8% of participants agreed that: ‘if an autistic child does not want to participate in a PA programme, they should first receive encouragement from staff and parents before being allowed to sit out’. Additional consensus statements related to the session content and set-up are presented in Table 3.

**Table 3.** Consensus Statements Relating to Session Content and Set up (Consensus ⩾67% Agreement).

Statement	Agreement (%)
Sessions should be a combination of games-based and sport-specific	81.25
Sessions should break down sport-specific exercises into smaller, more manageable skills	94.5
Community-based PA programmes which include autistic children should consist of a combination of individual activities and group activities	93.8
Programmes should be modelled around evidence-based research	83.4
Extra coaches/volunteers should be present to assist autistic children in these programmes to minimise disruption to non-autistic children	88.9
An autistic child should be given time to become comfortable with a new environment/setting before deciding they do not wish to participate (i.e., be brought to a few sessions before ceasing participation altogether)	94.5
An autistic child should be repeatedly encouraged to participate in PA programmes, even if they do not want to, because these programmes are important for their physical and mental health	81.25
Parents should be given the opportunity to highlight any needs, preferences or behavioural issues specific to their child to coaching staff prior to bringing them to an initial session	100
Parents of autistic children with behavioural problems must outline triggers and de-escalation plans to coaches before the child can join a PA programme	87.5

PA: physical activity.

Experts were asked how important they believed certain elements of PA are for autistic children. Notably, the development of FMS, improving balance and coordination, and having fun were all considered somewhat/very important by 100% of experts. Development of muscular strength, aerobic endurance, mobility or flexibility, and social aspects were considered somewhat/very important by 94.4% of experts.

#### Round 3

In Round 3, consensus was formed to indicate that PA programmes involving autistic children should predominantly work on developing FMS (87.6% agreement). Regarding intensity, 87.5% of experts considered it an essential factor of a PA programme, and 81.3% of experts thought programmes should aim to achieve a moderate-to-vigorous intensity, be in line with the American College of Sports Medicine recommended PA guidelines.

### Bridging Sessions

Bridging sessions refer to the concept of autistic children partaking in small-group sessions, which serve as an introduction to a mainstream PA programme. These sessions may focus on the development of motor skills and introducing relevant rules or skills, where the mainstream programme is for a specific sport. In Round 1, when asked about how we can support autistic children in community-based programmes, one expert highlighted that bridging sessions can be *‘platforms for onward integration into community PA and sport opportunities’.*

In Round 2, 88.9% of experts somewhat/strongly agreed that clubs should offer community-based ‘bridging sessions’ for autistic children before integration into mainstream PA sessions. Bridging sessions were further discussed in Round 3, with one expert noting that: *‘. . .bridging-sessions can begin to help establish structures and norms that may prepare them for future integration into a community sports group’*. Additional consensus statements from Round 3 are presented in Table 4.

**Table 4.** Consensus Statements Relating to Bridging Sessions (Consensus ⩾67% Agreement).

Statement	Agreement (%)
*Bridging sessions should . . .*	
. . . be offered to all autistic children	81.3
. . . double as a form of screening, to assess which mainstream session may be suitable	87.6
. . . focus on the development of FMS	91.67
. . . focus on the specific skills and rules necessary for a particular sport	81.3
. . . be a combination of both FMS and sport-specific skills or rules	87.6
. . . be tailored to the individual abilities of the children partaking	93.8
. . . be done in small-group settings, i.e., less than five children	75.1
Having an educated coach to lead bridging sessions is feasible in community-based settings	75.1
Transitioning into mainstream should be a collaborative decision between the coach, parent/caregiver and child	83.34

FMS: fundamental movement skills.

While there was an emphasis on integration into mainstream programmes, experts also agreed that ‘a community-based PA programme with non-autistic children is not suitable for an autistic child who displays problematic behaviours such as aggression, self-harm, destruction of equipment, tantrum, etc.’ (75% agreement) and ‘clubs/groups should offer PA programmes that specifically do not focus on winning but instead focus on equal participation and enjoyment to all children, not just those with disabilities’ (77.8% agreement).

## Discussion

This study aimed to collate the perspectives and opinions of three key stakeholder groups (academics, working professionals, and autistic individuals) to compile a series of best practice recommendations which could be used by clubs/organisations when setting up or modifying community-based PA programmes to be inclusive of autistic children. To our knowledge, this is the first study to use expert consensus in creating inclusion recommendations for autistic children in commnity-based PA programmes. From this Delphi process, a series of recommendations were established that related to three main themes: (1) coaches and volunteers, (2) programme characteristics, and (3) bridging sessions. Within these recommendations, a child-centred approach is always recommended (Muselaers et al., 2025). This involves recognising the heterogeneity of autistic children and tailoring programmes to the individual child’s needs, strengths, communication style, and interests for optimal engagement.

### Coaches and Volunteers

Experts agreed that the most common barriers to PA participation for autistic children were factors relating to coaches and the lack of autism-specific education available. Similarly, a scoping review by Okkenhaug et al. (2024), which looked at barriers and facilitators to PA for autistic children, highlighted that coaches/teachers’ competence in coaching autistic children while also mitigating bullying/exclusion by peers was commonly reported across the studies included. In addition, several of the included studies reported that coach/teacher relationships with autistic children are crucial in facilitating participation.

According to the consensus from the experts in this study, coaches working with autistic children should be educated in methods of inclusion, behavioural strategies, the use of the PECS, sensory integration, and the development of FMS in this population. A study by Ohrberg et al. (2013) also supports this consensus, reporting that while coaching staff are typically required to be educated in general coach education, a greater emphasis on autism-specific training would be needed to better support autistic children. For coach education to be meaningful and effective, courses should centre learning outcomes around up-to-date evidence-based research, rather than being *‘merely autism awareness-based’*, as one expert cited. Coaches working with autistic children have previously reported similar statements, highlighting the need for autism-specific coaching courses which focus on practical applications for coaches (Kimber et al., 2023). Similarly, Edwards et al. (2024) highlighted the need for autism-specific training not only for coaches but also volunteers – a finding which is supported by panellists of this study, who concluded that volunteers should also undertake autism-specific training.

While support needs vary among autistic children, experts agreed that a low coach-to-child ratio (at least 1:2) is critical to support autistic children in PA programmes. The issue then becomes the availability of coaches who have this specialised training. This issue is complex in that for coaches to be adequately trained to work with this population, they must first be motivated to complete training, and training opportunities must also be available. Previous research reported that coaches without prior experience of coaching athletes with disabilities were apprehensive about getting involved due to their perceived lack of knowledge/experience (Wareham et al., 2017). This may be a factor in coaches not upskilling or seeking out additional training. The Delphi panel identified that this training should be facilitated by clubs/governing bodies rather than being down to the volition of the individual coach to source appropriate training. Notably, it has been reported that community agencies often lack the qualified training personnel or revenue to fund specialised training (Glasgow & Emmons, 2007), which may suggest a need for governing bodies to facilitate this training.

Volunteers play a significant role in participant experience in community-based programmes (Man et al., 2021). While this Delphi study was inconclusive in developing exact ratio recommendations for the number of volunteers to children, the average answer was approximately 1:2. A ratio of 1:3/4 was previously used in a multi-sport programme for autistic children, in which 71% of participants had a positive experience (Rosso, 2016). However, the coaches involved in this study suggested a lower ratio of 1:2 would have enabled children to receive more constant attention from coaches. Notably, the parents of autistic children in another study suggested a preference for lower ratios, with a main head coach and a 1:1 volunteer-to-child ratio (Alexander & Leather, 2013). Unequivocally, the necessary coach/volunteer-to-child ratios will be dependent on the individual child. While recommendations for ratios can be made, a child-centred approach is still needed due to the diversity within this population. Regardless of the exact ratio, emphasis should be placed on creating meaningful and individualised coach–child relationships to facilitate long-term PA participation (Kimber et al., 2023).

### Programme Characteristics

Expert consensus found that individual PA sessions should be 30–45 minutes, twice weekly, for autistic children. A scoping review, which looked at school-based PA programmes for autistic children, found that interventions were generally run two to three times per week with 60 minutes being the most frequent session length (Campos et al., 2023). The shorter recommendation from this study may be advantageous for maintaining concentration and participation in autistic children. The duration of programmes was discussed in this study; however, several participants noted that programmes should not be run for a set number of weeks but rather should run indefinitely, becoming part of the child’s weekly routine, aiming to align with WHO guidelines of 60 minutes of moderate-to-vigorous intensity PA per day (WHO, 2020). In addition, very few PA intervention studies for autistic children include long-term follow-up assessments, which renders determining the long-term effects of participating in PA interventions on this population challenging (Ji et al., 2023).

The development of FMS was deemed to be an essential aspect of any PA programme. This aligns with research suggesting deficits in motor skills are prevalent in autistic children (Gandotra et al., 2020; Licari et al., 2022; Liu & Breslin, 2013; Whyatt & Craig, 2012). Dong et al. (2024) found motor deficits or signs of motor delays to be present in ~80% of autistic children, with motor skill scores being significantly less than in non-autistic peers. In addition, deficits in motor skills are frequently cited as a barrier to PA participation for this population (Must et al., 2014; Salar et al., 2024), a factor that Delphi panellists also noted. A focus on the development of motor skills is therefore a necessary component of all PA programmes that include autistic children and may help to reduce restrictions to PA participation for this population.

### Bridging Sessions

While the idea of bridging sessions was prevalent throughout this Delphi study, there was debate among the experts as to whether programmes in competitive environments are suitable for all autistic children. Competitive environments often do not align well with common characteristics associated with autism (Must et al., 2015; Okkenhaug et al., 2024). Experts in this study concluded that these environments are likely not suitable for children with higher support needs or those with challenging behaviours (e.g., aggression, self-harm or tantrums) and additionally agreed that clubs should offer non-competitive programmes for all children and not just those with disabilities. This builds on previous research, which suggests that children who have higher support needs likely needed individual sessions, while group settings may benefit those with lower support needs due to the added social aspect (Srinivasan et al., 2014). However, as with all recommendations, a child-centred approach should be taken rather than making generalisations.

### Limitations

Notwithstanding the novel information provided in this study, it is prudent to acknowledge certain limitations. The study attempted to compile a diverse expert panel to achieve a broad range of expertise and opinions. While all participants were considered experts in autism inclusion in sports, professions, backgrounds, and experiences were diverse within the group. This may have resulted in some experts being more/less knowledgeable on specific topics. In addition, there may have been differing opinions on some topics due to cultural differences, which are likely present among an international panel. However, while the previous points can be viewed as limitations, it is also a unique strength of the Delphi process. Diversity among the panel allowed for a broader range of opinions and perspectives, resulting in well-rounded consensus statements. In addition, while this study included the perspectives of autistic adults who had either experience coaching or playing sport themselves, identifying and recruiting participants from this population was particularly challenging, with only two autistic individuals being involved in the panel. However, the inclusion of autistic individuals within the panel did provide an important perspective, which is not commonly seen within the literature. Finally, while the Delphi panel aimed to include a broad range of experts, the views of autistic children and their parents were not included. Future research should aim to incorporate their perspectives to ensure the STARTS recommendations represent their lived experience.

## Conclusion

**Figure 2.**
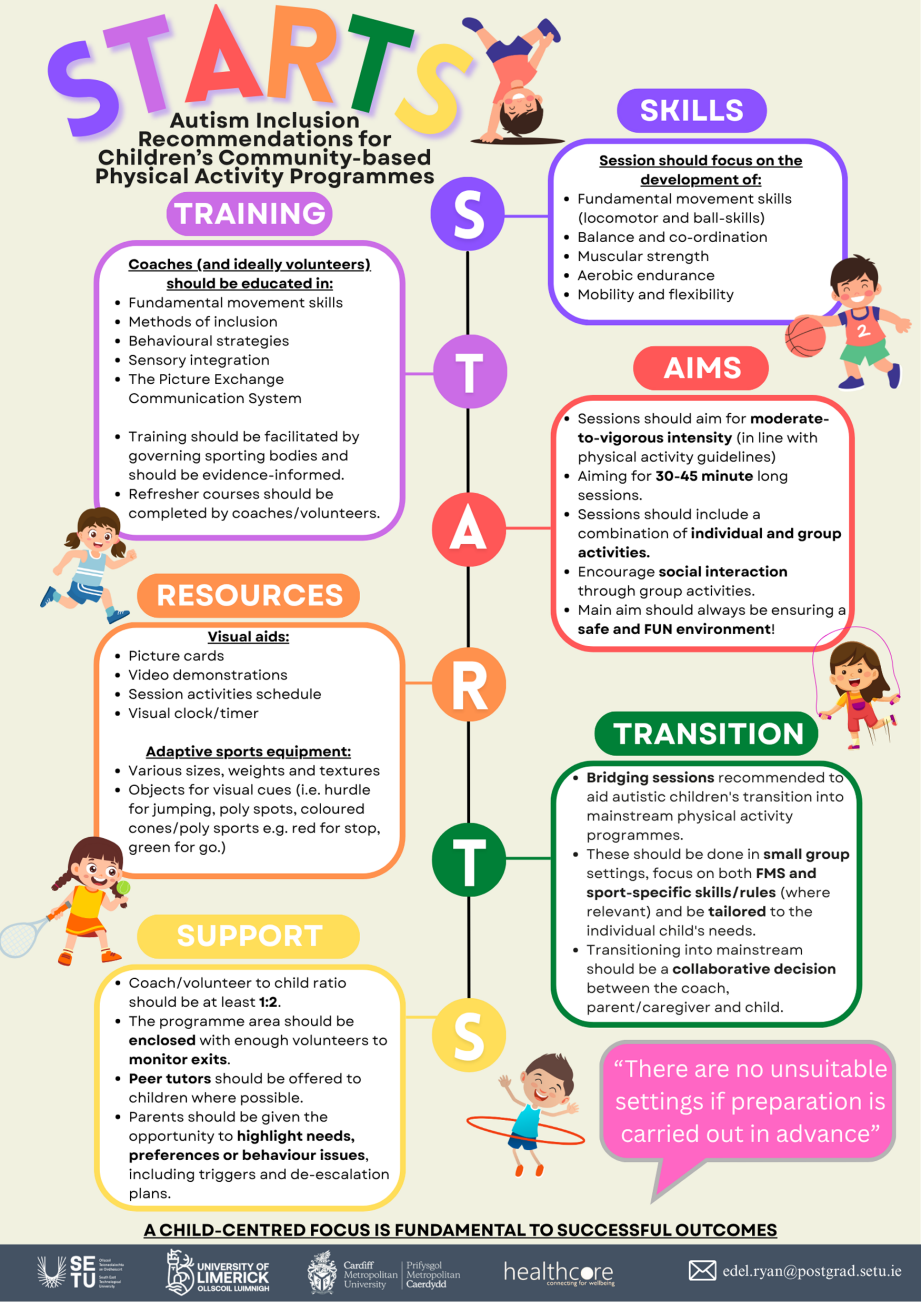
STARTS autism inclusion recommendations for children’s community-based physical activity programmes.

This Delphi study has presented a range of consensus statements relating to autism inclusion recommendations for community-based PA programmes. Recommendations pertaining to coaches and volunteers, programme characteristics and bridging sessions were developed, informed by the perspectives of academics, coaches, and autistic individuals. These consensus statements (Supplementary File 1) were then adapted into the ‘STARTS’ recommendations (Figure 2), an accessible infographic which breaks the recommendations down into (1) Skills, (2) Training, (3) Aims, (4) Resources, (5) Transition, and (6) Support. These recommendations are informed by expert consensus and offer a supportive framework for clubs/organisations to utilise when setting up or altering PA programmes, which may improve the inclusion of autistic children in these programmes. In addition, it can be used to inform future autism inclusion training for coaches/volunteers and help to shape the design and delivery of applied PA interventions for autism-based research projects. While this study achieved expert consensus, further research is required to examine the implementation and evaluation of the STARTS guidelines for feasibility and effectiveness in community-based settings. In addition, future research should focus on further exploring the concept of bridging sessions and how they may be implemented, as well as investigating the suitability of the STARTS recommendations for autistic children with higher support needs.

## Supplemental Material

sj-docx-1-aut-10.1177_13623613261448516 – Supplemental material for Original ArticleRecommendations for the Inclusion of Autistic Children in Community-Based Physical Activity Programmes: A Delphi Study Supplemental material, sj-docx-1-aut-10.1177_13623613261448516 for Original ArticleRecommendations for the Inclusion of Autistic Children in Community-Based Physical Activity Programmes: A Delphi Study by Edel Ryan, Dean McDonnell, Sean Healy, Rhodri S. Lloyd and Sharon Kinsella in Autism

sj-docx-2-aut-10.1177_13623613261448516 – Supplemental material for Original ArticleRecommendations for the Inclusion of Autistic Children in Community-Based Physical Activity Programmes: A Delphi Study Supplemental material, sj-docx-2-aut-10.1177_13623613261448516 for Original ArticleRecommendations for the Inclusion of Autistic Children in Community-Based Physical Activity Programmes: A Delphi Study by Edel Ryan, Dean McDonnell, Sean Healy, Rhodri S. Lloyd and Sharon Kinsella in Autism
