# Supplementary material for: Recommendations for the Inclusion of Autistic Children in Community-Based Physical Activity Programmes: A Delphi Study
Source: Autism. 2026 May 31;30(8):1986–99. doi: 10.1177/13623613261448516 (PMC13392180; doi:10.1177/13623613261448516)
Supplement: sj-docx-1-aut-10.1177_13623613261448516 – Supplemental material for Original ArticleRecommendations for the Inclusion of Autistic Children in Community-Based Physical Activity Programmes: A Delphi Study [file sj-docx-1-aut-10.1177_13623613261448516.docx]

| Coaches and Volunteers |
| --- |
| 1. Coaches should be educated in the following areas:    1. FMS;    2. Methods of inclusion;    3. Behavioural strategies;    4. Sensory integration; and    5. The Picture Exchange Communication System. 2. Volunteers should ideally be educated in the same areas as coaches. 3. This training should be facilitated by the management of clubs and governing sporting bodies. 4. Coaches/volunteers should be completing refresher courses in the above-mentioned areas. 5. Where one educated coach is present, it is recommended that the volunteer-to-child ratio be 1:2. |
| Programme Characteristics |
| 1. Recommended resources:    1. visual aids    2. sensory break areas    3. timer    4. adaptive sports equipment. 2. Session duration: 30–45-minute sessions. 3. Intensity: aiming for moderate-to-vigorous 4. Health and safety: The programme area should be enclosed with enough volunteers to monitor exits. 5. Session focus:    1. Developing FMS, balance, coordination, muscular strength, aerobic endurance, mobility and flexibility through individual and group activities;    2. Encouraging social interaction with group activities;    3. All while ensuring children are having fun. 6. It was agreed that programme design should be evidence-informed however, it was highlighted that coaches currently do not have adequate and appropriate access to research. It is suggested that sports governing bodies should provide up-to-date resources to better inform their coaches in best practice guidelines. 7. Parents should be given the opportunity to highlight needs, preferences or behaviour issues, including triggers and de-escalation plans. 8. ‘Peer tutors’ should be offered to children where possible. |
| Bridging Sessions |
| 1. Bridging sessions are recommended to aid in autistic children's transition into mainstream PA programmes. 2. These should be done in small group settings, focus on both FMS and sport-specific skills (where relevant) and be tailored to the individual child's needs. 3. Transitioning into mainstream should be a collaborative decision between the coach, parent/caregiver and child. |
