# Supplementary material for: Recommendations for the Inclusion of Autistic Children in Community-Based Physical Activity Programmes: A Delphi Study
Source: Autism. 2026 May 31;30(8):1986–99. doi: 10.1177/13623613261448516 (PMC13392180; doi:10.1177/13623613261448516)
Supplement: sj-docx-2-aut-10.1177_13623613261448516 – Supplemental material for Original ArticleRecommendations for the Inclusion of Autistic Children in Community-Based Physical Activity Programmes: A Delphi Study [file sj-docx-2-aut-10.1177_13623613261448516.docx]

*The following document outlines the open-ended questions which were featured across the three rounds of the Delphi. The responses to these questions were thematically analysed following the methods outlined in the manuscript.*

**Round 1**

Q 1:

1. What do you believe to be the most significant issues which hinder the inclusion/participation of autistic children in community-based sport and exercise programmes? Please list up to five (in no particular order)
2. Do you have any suggestions as to how these could be addressed? Please elaborate on your answers.
3. Please leave any additional comments you may have for the above questions.

Q 2:

1. Please list up to five factors/considerations you believe to be most important for supporting the participation of autistic children in community-based sport and exercise programmes (in no particular order):
2. Do you think these factors/considerations are, in your experience, well implemented/utilised in sport and exercise programmes? Please elaborate on your answers
3. Please leave any additional comments you may have for the above questions.

Q 3:

1. Who do you think the key stakeholders/individuals that can facilitate participation of autistic children in physical activity programmes? Please elaborate where possible.

Q 4:

1. What do you think makes a community-based setting suitable/not suitable for an autistic child?
2. Do you have any examples of community-based physical activity programmes which successfully or unsuccessfully included autistic children? What defined these programmes?

Q 5:

Participants were provided with three case-vignettes of autistic children with various support needs and asked the following questions:

1. Do you think community-based physical activity programmes with non-autistic children would be a suitable environment for this child?
2. What do you think might hinder this child’s participation in community-based sports programmes?
3. What adaptations would you recommend to facilitate the inclusion of this child in community-based programmes?

Q 6:

1. Please use the space below to detail any other comments, ideas or suggestions regarding the inclusion of autistic children in community-based physical activity programmes.

**Round 2:**

1. If there is anything ese you would like to mention regarding coach education, please use the space below.
2. Do you have any examples/types of games/activities that, in your experience, are well received by autistic children?
3. Do you have any examples/types of games/activities that, in your experience, are not well received by autistic children?
4. If there is anything else you would like to mention regarding programme characteristics, please use the space below.
5. If there is anything else you would like to mention regarding stakeholders/facilitators, please use the space below.
6. Autistic children often need breaks for physical activity or may not choose to participate. Do you have any suggestions as to how to encourage children to participate/re-join a game after a break?
7. Some autistic children can pose as a ‘flight risk’ (i.e. can run off without warning). What strategies would you suggest to minimise risk for these children?
8. If there is anything else you would like to mention in terms of child-related barriers, please use the space below.

**Round 3:**

*Following on from questions/items regarding motor skills:*

1. Do you/did you ever assess a child’s fundamental movement skills and what method do you/did you use?

*Following on from questions/items regarding intensity of sessions:*

1. If you selected that you think intensity is an important factor, do you think monitoring intensity levels is also important and do you have any suggestions/recommendation as to how you would monitor intensity levels?
2. If there is anything else you would like to mention in regard to any of the topics discussed in this round, please use the space below.
